# Investigating and Correcting Plasma DNA Sequencing Coverage Bias to Enhance Aneuploidy Discovery

**DOI:** 10.1371/journal.pone.0086993

**Published:** 2014-01-29

**Authors:** Dineika Chandrananda, Natalie P. Thorne, Devika Ganesamoorthy, Damien L. Bruno, Yuval Benjamini, Terence P. Speed, Howard R. Slater, Melanie Bahlo

**Affiliations:** 1 Bioinformatics Division, The Walter and Eliza Hall Institute of Medical Research, Melbourne, Australia; 2 Department of Medical Biology, University of Melbourne, Melbourne, Australia; 3 Victorian Clinical Genetics Services Cytogenetics Laboratory, Murdoch Childrens Research Institute, Melbourne, Australia; 4 Department of Paediatrics, University of Melbourne, Melbourne, Australia; 5 Department of Statistics, University of California, Berkeley, California, United States of America; 6 Department of Mathematics and Statistics, University of Melbourne, Melbourne, Australia; Shenzhen Institutes of Advanced Technology, China

## Abstract

Pregnant women carry a mixture of cell-free DNA fragments from self and fetus (non-self) in their circulation. In recent years multiple independent studies have demonstrated the ability to detect fetal trisomies such as trisomy 21, the cause of Down syndrome, by Next-Generation Sequencing of maternal plasma. The current clinical tests based on this approach show very high sensitivity and specificity, although as yet they have not become the standard diagnostic test. Here we describe improvements to the analysis of the sequencing data by reducing GC bias and better handling of the genomic repeats. We show substantial improvements in the sensitivity of the standard trisomy 21 statistical tests, which we measure by artificially reducing read coverage. We also explore the bias stemming from the natural cleavage of plasma DNA by examining DNA motifs and position specific base distributions. We propose a model to correct this fragmentation bias and observe that incorporating this bias does not lead to any further improvements in the detection of fetal trisomy. The improved bias corrections that we demonstrate in this work can be readily adopted into existing fetal trisomy detection protocols and should also lead to improvements in sub-chromosomal copy number variation detection.

## Introduction

Detection of trisomy 21, also known as Down syndrome, has long been considered the driving force for prenatal diagnosis. This disorder, which causes severe intellectual and developmental disability, is the most common fetal chromosomal defect with a prevalence of 1 in 700 newborns [1]. Other aneuploidy conditions such as trisomy 13 (Patau Syndrome) and trisomy 18 (Edwards Syndrome) are more lethal in infants but are much less frequent [2], [3]. Efforts to develop non-invasive prenatal tests (NIPT) for detection of these chromosome abnormalities have been spurred on by the increasing maternal age in developed countries and the associated increase in the risk of fetal aneuploidy [4], [5].

The 1997 discovery of the existence of cell-free fetal DNA in maternal plasma [6] provided a new avenue for non-invasive prenatal testing. A decade and a half of concentrated research efforts coupled with the recent rapid advances of Next-Generation Sequencing (NGS), have now allowed sequencing based aneuploidy tests to be clinically translated in several countries including USA and China. Since the initial proof-of-principle studies in 2008 [7], [8], the NGS platform type, sequencing scope and depth used in NIPT have been rapidly changing, in the quest for greater sensitivity of trisomy detection and the need to investigate sub-chromosomal copy number changes in the fetus in the most cost-effective manner [9], [10].

### Characteristics and biology of cell-free fetal DNA

Nucleic acids can be found in cell-free form in human plasma and serum [11]. This DNA has recently been demonstrated to be predominately of hematopoietic origin [12] and apoptosis has consistently been shown as a major source of this cell-free DNA (cfDNA) [13]–[15]. However, other biological sources have also been implicated and there remains uncertainty in the field as to the involvement of various processes such as the active secretion of cfDNA by cells and the role of membrane proteins in protecting cfDNA in circulation [16], [17].

In early pregnancy, 3–20% of the DNA in maternal plasma comes from the developing fetus [18]–[20] and this proportion is shown to increase with gestational age [21], [22]. An inverse relationship has been described between the fetal proportion and maternal weight, indicating a possible dilution effect [23], [24]. Fetal cfDNA is detectable from the 7th week of gestation and the most likely source is thought to be apoptosis of placental (i.e. extra-embryonic) cells [25], [26].

Paired-end sequencing of plasma DNA has revealed that the fragments are generally around 166 bp in size with a small proportion exhibiting a length close to 340 bp [27]. The major peak in the size distribution is very similar to the length of DNA that is wrapped around a nucleosomal core unit (approximately 146 bp plus a linker fragment of DNA between 20–90 base pairs [28]). The 340 bp signal corresponds to a di-nucleosomal structure. The same study showed that fetal fragments were generally shorter than 150 bp.

### NGS-based Down syndrome detection

While a few clinically available tests use targeted sequencing to select DNA fragments from specific chromosomes [29], [30], the majority currently utilize a genome-wide sequencing protocol that has been shown to be reproducible in multiple studies [24], [31]–[33]. The largest clinical validation studies that use whole-genome sequencing have all used variations of the “molecular-counting” approach.

This approach attempts to identify an increase in DNA fragments originating from the aneuploidy chromosome in the fetus without distinguishing between maternal and fetal DNA, using either lab-based or bioinformatics methods. The tests calculate the relative amounts of plasma DNA fragments originating from the different chromosomes and determine if there is an increase beyond what is expected for a euploid pregnancy assuming that this increase stems from a copy number change in the fetus. In a pregnancy with fetal trisomy 21, there would be a slight increase in the amount of DNA derived from chromosome 21 in maternal plasma compared with the DNA from other chromosomes. The extent of this increase is dependent on the fetal DNA proportion, in that, if fetal DNA constitute 10% of the ccfDNA in a pregnancy with fetal trisomy 21, we would anticipate a 5% increase in the copy number of chromosome 21 fragments in maternal plasma. This can be assessed by comparing the chromosome 21 read counts for an unspecified pregnancy to that of a set of controls (euploid pregnancies).

### Biases in sequencing data

Since the tests attempt to detect small increases in the chromosomal representation, they incorporate steps in their analysis that adjust for the bias inherent in NGS data which lead to inter- and intra-chromosomal read count variability.

One such bias is the Guanine-Cytosine content (GC) bias which is uni-modal in nature as the read coverage is maximized for genomic regions with 40–50% GC with coverage decreasing at the extreme values. The current tests predominantly incorporate a locally weighted scatterplot smoothing (LOESS) correction step as described by Alkan et al. [34] to correct for GC. This involves binning the read counts into non-overlapping windows and calculating the GC content of these windows (a 50 Kb bin size is in wide use). A LOESS curve is fitted to the plot of bin counts vs. bin GC to obtain predicted values that are used to correct the raw counts.

A second source of bias stemming from the alignment of short read data is the inability of sequence reads to map unambiguously or uniquely to highly repetitive regions of the genome. This mappability bias is generally dealt with by removal of such regions (annotated in the RepeatMasker database [35]) rather than quantifying the mappability and correcting for it.

### Statistical test for trisomy

Different research groups use different algorithms to test for aneuploidy in the bias corrected data. Since they generally have access to large sample sizes of both normal and aneuploid pregnancies they are able to make use of these samples to perform an empirical bias adjustment, per chromosome, in their statistical analysis [31], [36].

### Motivation

In this work, we present a post-sequencing protocol that utilizes a more sensitive GC correction (based on the work of Benjamini and Speed [37]) as well as a correction for mappability. We also show an additional source of bias due to fragmentation that is inflated in plasma cfDNA when compared to genomic DNA. The fragmentation effect is the position-specific pattern of nucleotides around DNA fragment ends. Plasma DNA shows different and stronger patterns than genomic DNA due to the fragments originating from a biological process and possible nuclease activity rather than a random shearing during the DNA fragmentation step of library preparation. This bias is investigated in depth to illustrate its relationship with the GC bias. We then extend the improved GC correction to incorporate the fragmentation bias.

The motivation of our work is to compare between different bias correction protocols in order to recommend an optimal correction procedure. Since optimization of the test statistic can be considered a separate research area, we use the simple Z-score method described by Chiu et al. in 2008 and 2011 [7], [19], to benchmark the results of our correction methods. Furthermore, the Z-score method has been the basis of many of the subsequent tests so we used this test statistic for our comparisons. We find that considerable improvements in trisomy detection are achieved using our recommended pipeline.

## Materials and Methods

### Ethics Statement

This study was approved by the Royal Children's Hospital (RCH) Human Research Ethics Committee (HREC). All participants gave written informed consent to the protocol approved by the RCH HREC (reference number 31080A).

### Subject recruitment and clinical information

Pregnant women in their first and second trimester of pregnancy were recruited through the Victoria Clinical Genetics Service (VCGS) after providing informed written consent. All participants donated 10 mL of peripheral blood. Samples used in this study were collected prior to any invasive testing and all were from singleton pregnancies.

Two patient groups were included: The first were women who had received a post-test low-risk score by Combined First Trimester Screening. Samples from these women were assumed as euploid and used as ‘normal’ controls; pregnancy outcomes were consistent with categorizing these as ‘likely’ euploid. The second were women with a diagnosis of fetal trisomy 21, which was made by chromosome analysis of amniocentesis or chorionic villus samples.

### Processing of blood samples

The collected blood samples were stored in tubes containing Ethylene Diamine Tetraacetic Acid (EDTA) and were processed within 4 hours after phlebotomy by a double-centrifugation protocol. In brief, blood was transferred into 15 mL falcon tubes and centrifuged at 1600 g for 10 minutes at 4°C to separate the blood cells and plasma. Next, plasma was transferred into 1.5 mL micro-centrifuge tubes and centrifuged at 16000 g for 10 minutes at 4°C to remove residual cells. Plasma was transferred into new microcentrifuge tubes and stored at −80°C until further processing. The blood cell fraction was stored at −20°C until further processing.

### DNA library preparation and sequencing

DNA library preparation was carried out at the Murdoch Childrens Research Institute where the samples were de-identified and assigned anonymous IDs for the purposes of this study.

Cell-free DNA was isolated from plasma using the QIAamp Circulating Nucleic Acid Kit (Qiagen, Melbourne, Australia) according to the manufacturer's guidelines, with slight modifications in reagent volumes.

DNA libraries were prepared according to a modified protocol from Illumina (http://www.illumina.com/). After end-repairing, A-base tailing and adaptor ligation, standard multiplex primers were introduced by PCR with 5 mL of PCR Primer Cocktail and 25 mL of PCR Master Mix. The PCR protocol consisted of a denaturation step at 98°C for 30 seconds and 10 cycles of 98°C for 10 seconds, 60°C for 30 seconds and 72°C for 30 seconds and final extension at 72°C for 5 minutes and held at 10°C.

The size distribution of the libraries was analyzed using an Agilent Bioanalyzer and quantified with real-time PCR. The fragmentation and size selection steps in the library preparations were omitted, as the plasma DNA molecules are naturally cleaved and exist as short fragments [27].

All samples were sequenced at the Australian Genome Research Facility on the Illumina HiSeq 2000 platform. Barcoded libraries were equally pooled in batches of 6 in one lane and sequenced with a 50-cycle single-end multiplex strategy. Thirty libraries were sequenced in total using the 6-plex format in five batches. The batches were run at different times and using different flow-cells.

### Sequenced samples

Initially, blood samples from 24 pregnant women were used to make 24 DNA libraries. All samples, except one, had clinical information available on the Combined First Trimester Screening test. The single sample that was missing this information was removed from further analyses leaving 23 samples.

Six of the remaining libraries were re-amplified using either 3 or 5 cycles of PCR based on the library yield, in order to act as technical replicates in the study. Hence, twenty-nine 50 bp single-end read datasets were utilized in the subsequent analysis. Of these, 9 were from trisomy 21 cases and 20 were normals. All 29 read datasets are accessible through the Short Read Archive (http://www.ncbi.nlm.nih.gov/Traces/sra/) under the accession number SRA097799.

### Sequencing data processing

Following sequencing, reads were stripped of their adapters and demultiplexed according to their barcodes. The reads for the 29 samples were then aligned to the human genome, build 37 (hg19) using Novoalign V2.08.03 (www.novocraft.com). An ambiguous human reference was used that has known SNPs encoded as IUPAC ambiguous codes. Reads that mapped to multiple-locations, those that were designated as PCR duplicates as well as reads that aligned with more than two mismatches were discarded from each dataset using a combination of Samtools V0.1.18 [38] and Picard software V1.65 (http://picard.sourceforge.net/).

Each sample, consisting of a set of reads from an individual, had the following four bias correction protocols applied to allow comparisons in their performance. All the following analyses were performed in R [39].

1. RM_LOESS: reads that fall into repeatMasker regions (http://www.repeatmasker.org) are removed and a LOESS correction performed for GC bias (using the ‘loess’ R package).
2. mapCorr_LOESS: read counts are corrected according to the mappability [40] of the regions and a LOESS correction performed for GC bias.
3. mapCorr_singlePos: GC bias was estimated and corrected via the single position model [37] after correcting read counts according to the mappability of regions.
4. mapCorr_singlePos_Frag: As in 3) but corrections carried out after stratifying reads into classes based on their fragmentation signature.

Method 1 is a commonly used bias correction approach in the NIPT field, whilst methods 2)-4) represent new protocols.

In order to assess the robustness of the bias corrections, the read alignment of the 29 sequencing datasets was repeated with a different aligner, BWA (V 0.7.2-r351) [38], which uses an algorithm different to that of Novoalign. BWA is freeware and is commonly used by other researchers to align NGS data. Its algorithm is unable to utilize the ambiguous reference; hence the sequencing reads were mapped to the human genome with unmasked SNPs.

### LOESS GC bias correction

The LOESS correction performed was similar to the one described by Alkan et al. 2009 [34]. The genome was segmented into non-overlapping bins of 50 Kb length and the chromosomal read coverage as well as GC content of each bin was calculated (chrY was excluded from this analysis). Bin counts are the total number of reads with the 5′ end inside the bin. GC content is the proportion of guanine and cytosine bases in the bin as per the reference genome. After filtering regions with no counts, the average read count for windows with GC content in intervals of 1% were calculated. A LOESS regression curve was fitted to this data with a span of 0.3, to determine a predicted count for each bin along the genome based on its GC content using the loess function in R. The read count of each bin was normalized by the predicted bin count to obtain the corrected value.

Fifty kb bins were used in the analyses as it is the most widely used bin size in the NIPT literature. The choice for this bin size was stated to be arbitrary in an early proof of principle publication [41].

### Usage of Repeat Masker

Reads that were mapped to repeat-masker annotated sites (Repeat Library 20120124; http://www.repeatmasker.org) were discarded prior to GC correction.

### Mappability correction

Short sequence reads were simulated by fragmenting the human reference genome (hg19) into overlapping fragments at one base-pair resolution. The length of the simulated reads was set to 50 bp, as this was the read length in the sequencing data. The reads were aligned back to the reference genome using Novoalign, and all base positions of uniquely mapping reads were identified. The proportion of ‘unique’ sites in the window then gives its mappability value.

The read counts in a given window were multiplied by the reciprocal of its mappability value in order to correct for this mapping bias in the sequencing data. Fifty Kb windows with less than 50% unique sites were filtered out to prevent over-correction. Data aligned using BWA were corrected with mappability annotation created using BWA.

### Single-position GC bias estimation with mappability correction

Benjamini and Speed described a single-position GC bias correction model [37] and showed that it removed more bias than the commonly used LOESS method that is based on modeling read counts and GC content in non-overlapping genomic windows. The single-position model stratifies uniquely mappable positions along the genome by the GC content of the DNA fragment of fixed length beginning at the position. It then estimates a mean fragment rate for all positions with the same GC. The following steps summarize how this model was utilized in this study. All steps were carried out using the GCcorrect R package. (http://www.stat.berkeley.edu/~yuvalb/YuvalWeb/Software.html).

- Uniquely mappable positions along the genome (denoted by 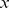) were randomly sampled. Each position was assigned a value corresponding to the number of GC bases in a window of length 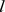 starting at position 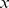. This was done in a strand-specific manner.
- Positions of similar GC value were classed into different GC strata. The total number of positions that fall into a certain stratum 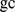 was denoted by 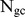.
- The number of DNA fragments 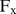 in each position 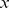 were counted so that the total number of fragments in each GC stratum 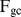 could be calculated.
- Mean fragment rates 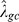 were estimated for each GC stratum by taking the ratio between fragment count 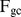 and positions 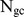. The plot of mean rates against GC was smoothed to obtain the predictions 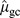 for each stratum.
- Since the GC stratum of each position 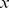 was known, the prediction per position 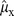 could be found using 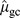.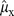 was equal to 0 if the position 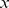 was not uniquely mappable.
- The read counts and predictions were aggregated separately into non-overlapping genomic windows of 50 Kb after the predictions per position were calculated in order to compare with the LOESS correction protocol. The counts were normalized by the aggregated predictions per window and multiplied by the reciprocal of the mappability value of the window as described before.

### Estimating the best GC window size

Benjamini and Speed found that the GC window size that captures the most variation was comparable to the length of the DNA fragments in the library. They recommended the use of the median fragment length of a sample. However, paired-end information was not available in the libraries used in this study so the fragment sizes could not be determined directly. Hence, the Total Variation from Independence score (TV-score) described by Benjamini and Speed was employed to find the optimal GC window.

The TV-score is a measure of how much of the variation in read depth is explained by conditioning on GC. Since it was known that the majority of plasma DNA fragments are shorter than 500 bp, the TV-score was calculated for GC-window sizes from one bp up to 700 bp. For each sample, the GC window that gave a high TV score and was comparable to fragment lengths of plasma DNA was used in the single position model.

### Investigating the fragmentation bias in plasma DNA

In their work, Benjamini and Speed examined the fragmentation bias present in genomic DNA and investigated the effect of this bias on the coverage. They concluded that in the genomic data the bias due to fragmentation was not significant, whilst GC content of the fragments was an important factor in determining coverage. We investigated fragmentation effects in this study due to the possibility that fragmentation bias plays a more important role in cell-free DNA given its exposure to non-random, biological processes such as apoptosis, which could result in fragmentation bias effects.

The following investigations were carried out in all 29 samples using stringently mapped reads (mapping quality greater than 60) that aligned to the 5′ and 3′ strands separately. The proportions of each nucleotide (A, T, C, G) in an interval surrounding read starts were calculated to examine any perturbations in the nucleotide proportions at the sites of DNA fragmentation.

Subsequently, the TV-score statistic was used to find which positions at the cleavage site were most influential in the fragmentation bias in order to reduce the fragmentation signature involving more than 18 positions. It should be noted that the TV-score is not specific to quantifying how much of the variation in read coverage is explained by the GC content of a window hence could also be used for this purpose. Reads were stratified according to the type of base at different positions surrounding the read starts and the fragment rates in these strata were used to calculate TV scores. The TV score in this setting measures how much variation in the read coverage is explained by conditioning on the base at each position. The positions that gave a high value relative to the others were determined visually and were aggregated. Thus, reads were stratified according to the k-mer at two or more positions instead of at a single position. This process was repeated until there was no visible increase in the TV-score and resulted in a simplified model for the bias by describing a motif consisting of the second base before the fragment starts as well as the first two bases into the fragment (positions -2,0,1), other bases did not impact the TV score once these bases were included in the model. The motif of nucleotides at these positions for a given read will henceforth be referred to as its breakpoint 3-mer. For example, C*CC is the breakpoint 3-mer corresponding to C at the −2,0 and 1 positions around the breakpoint with any of the four bases allowed at the −1 position.

Four nucleotides at three positions about the breakpoint result in a possible 64 combinations (4^3^) of breakpoint 3-mers. The proportions of all 3-mers were assessed for the 29 samples to detect any motifs that were consistently over-represented.

For both strands the frequency of reads for the common top twenty 3-mers was calculated and the rest were aggregated into a single class. In order to assess any strand specific fragmentation signature, a chi-square test for homogeneity was carried out to compare the proportions of the two multinomial distributions.

### Incorporating fragmentation effect into the single-position model

The fragmentation bias can be corrected by applying read stratification by motif. Reads were stratified according to the breakpoint 3-mer and the single-position GC correction was carried out within each separate stratum. Stratifying reads from low-coverage datasets into 64 classes creates too many strata with low counts of fragments; hence the strata were pooled into two classes that were determined by examining the GC profiles of fragments in the strata as well as the marginal frequency distributions.

With this stratification, the single-position model produces two predicted counts for every 50 Kb genomic bin. To correct the count in a bin, its observed counts for each stratum were normalized by their respective stratum predictions. The corrected stratum bin counts were aggregated within a bin using the proportion of reads belonging to the two strata as weights.

### Test for trisomy

Bias corrected sequence counts in genomic windows were used to calculate a standard Z-test of proportions as described by Chiu et al. [19]. The chromosome 21 proportion metric 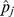 was computed for each sample, as the total bin count for chromosome 21 divided by the sum of the bin counts for all chromosomes except for the Y chromosome. The Z-score was defined as,
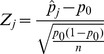
where 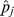 is the chromosome 21 proportion for sample *j* and 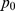 is the mean of the chromosome 21 proportions for the 20 non-trisomy samples (reference set, n = 20). A Z-score value of greater than 3 was chosen as a threshold to determine rejection of diploidy. This represents a chromosome 21 proportion greater than that of the 99.9th percentile of the reference set under the assumption of diploidy for a one-tailed Normal distribution.

### Evaluation of the bias correction algorithms

The performance of the different bias correction protocols was assessed by evaluating their ability to detect the known trisomy samples as the depth of sequencing was artificially reduced. For this purpose, aligned reads in each of the 29 datasets were separately sampled without replacement to generate different proportions to simulate data of lower coverage. The proportions used were 0.75, 0.5, 0.25, 0.125, 0.0625 and 0.0312. For each sample, the sub-sampled reads were processed as described previously and the sequence data at the 7 different coverage levels (original coverage as well as the 6 lower coverage levels) were run through the four different bias correction protocols.

Firstly, the data processed using the algorithms RM_LOESS and mapCorr_LOESS was used to compare the effect of the two methods for handling repeat regions in the genome. A LOESS smoother is separately fit to the data after each repeat handling protocol was carried out. The performance of the two methods is assessed by calculating the mean of the absolute difference between the observed fragment count and the LOESS prediction in each 50 Kb genomic bin. This estimate is known as the Mean Absolute Deviation (MAD). The MAD allows the comparison of the prediction error of the two LOESS models.

Secondly, the Z-score test was performed and the accuracy of trisomy detection between all four protocols was assessed. The difference between the 95th percentile of the normal chromosome 21 Z-scores and the 5th percentile of trisomy Z-scores (known as the discriminatory distance in NIPT literature) was calculated at each coverage level, as an additional method for comparison of the protocols.

Once the most sensitive bias correction algorithm was identified, an investigation was carried out to determine if this protocol could detect trisomy in a reference free setting to mimic the situation where no reference pool was available and to investigate the necessity of using the Z-score. This was done using methodology similar to the work of Fan and Quake [41], where the corrected bin counts of each chromosome were compared to all other chromosomes within the sample in order to detect over-representation. The details of this analysis are provided in Text S1.

## Results

Clinical details and sequence read statistics for the plasma DNA samples from 29 women are specified in Table 1. The gestational age of the pregnancies at the time of phlebotomy ranged from 9 to 16 weeks. Six-plex format, 50 bp single-end sequencing on the Illumina HiSeq 2000 platform generated a total number of reads ranging from 24.8 million to 40.3 million (an average of 32.1 million reads per sample). Novoalign was able to align an average of 88% of the total reads to the ambiguous reference while BWA mapped 86% to the non-ambiguous reference.

**Table 1:** Clinical details and sequencing data statistics of recruited samples.

Sample Index	Sample ID	Fetal Karyotype	Total Sequence Reads Generated (HiSeq 2000)	Reads Uniquely Aligned (Novoalign)	Reads Uniquely Aligned (BWA)
1	NIPD-03_TM	47, XX, +21	31,081,981	26,236,069	25,199,455
2	NIPD-03_TMR*	47, XX, +21	32,282,282	28,346,871	27,521,416
3	NIPD-04_TM	47, XY, +21	40,278,804	35,421,320	34,594,910
4	NIPD-04_TMR*	47, XY, +21	38,031,790	33,324,563	32,436,346
5	NIPD-05_TM	46, XX	33,885,622	29,897,378	29,186,676
6	NIPD-05_TMR*	46, XX	31,936,055	28,391,920	27,739,819
7	NIPD-07_TM	47, XY, +21	24,767,047	20,878,567	20,050,937
8	NIPD-07_TMR*	47, XY, +21	24,955,598	21,922,674	21,287,065
9	NIPD-09_TM	46, XY	26,987,293	23,723,020	23,164,487
10	NIPD-09_TMR*	46, XY	31,019,583	27,134,946	26,400,672
11	NIPD-13_TM	46, XY	28,932,490	25,534,678	24,925,205
12	NIPD-13_TMR*	46, XY	32,691,501	29,063,410	28,394,889
13	NIPD-48_TM	46, XY	31,048,049	27,414,008	26,758,810
14	NIPD-51_TM	46, XY	31,303,893	27,363,033	26,587,761
15	NIPD-54_TM	46, XX	32,519,963	28,835,431	28,135,842
16	NIPD-56_TM	46, XY	32,568,205	28,658,808	27,943,150
17	NIPD-58_TM	46, XY	28,960,279	25,087,238	24,309,884
18	NIPD-60_TM	47, XY, +21	32,137,330	28,409,821	27,687,323
19	NIPD-50_TM	47, XY, +21	33,458,803	29,815,550	29,158,995
20	NIPD-52_TM	46, XY	30,801,889	27,449,087	26,835,869
21	NIPD-53_TM	46, XX	33,135,305	29,454,208	28,740,525
22	NIPD-59_TM	46, XX	33,321,758	29,621,383	28,948,308
23	NIPD-61_TM	46, XX	38,287,043	34,235,072	33,502,284
24	NIPD-62_TM	46, XY	33,183,117	29,674,630	29,047,689
25	NIPD-23_TM	46, XX	29,852,910	26,152,316	25,492,998
26	NIPD-30_TM	46, XX	32,038,077	28,463,745	27,840,119
27	NIPD-63_TM	46, XX	32,085,733	28,679,366	28,097,170
28	NIPD-65_TM	46, XY	35,094,568	31,339,712	30,653,822
29	NIPD-66_TM	47, XY, +21	35,411,652	31,237,019	30,450,668

* Sample IDs with the suffix TMR belong to the re-amplified libraries.

Table 2 provides coverage statistics for the 29 samples after the raw reads were processed (aligned, filtered for multi-mapping, duplicates and mismatches). At the original sequencing depth, the samples exhibited average genome wide fold coverage of 0.42X (26 million reads) using Novoalign. The coverage was gradually reduced, through subsampling of the aligned data based on the Novoalign alignment, until the number of reads was comparable to the amount generated on an Illumina MiSeq sequencer with read lengths of 50 bp (proportions 0.125–0.0312 leading to coverage of 0.053X – 0.014X). The complementary statistics for the data aligned by BWA is given in Table S1.

**Table 2:** Genome-wide coverage at each sampling proportion for the 29 datasets after Novoalign mapping and subsequent read filtering.

Proportion of reads sampled	Average number of reads	Range	Mean fold coverage
original	26,174,727	18,832,054–32,733,023	0.42X
0.75	19,719,795	14,172,672–24,677,526	0.31X
0.5	13,206,439	9,483,101–16,539,568	0.21X
0.25	6,635,982	4,761,496–8,317,675	0.11X
0.125	3,328,165	2,387,989–4,173,351	0.053X
0.0625	1,667,889	1,196,946–2,091,602	0.026X
0.0312	854,222	613,083–1,071,927	0.014X

### Assessment of mappability bias

**Figure 1:**
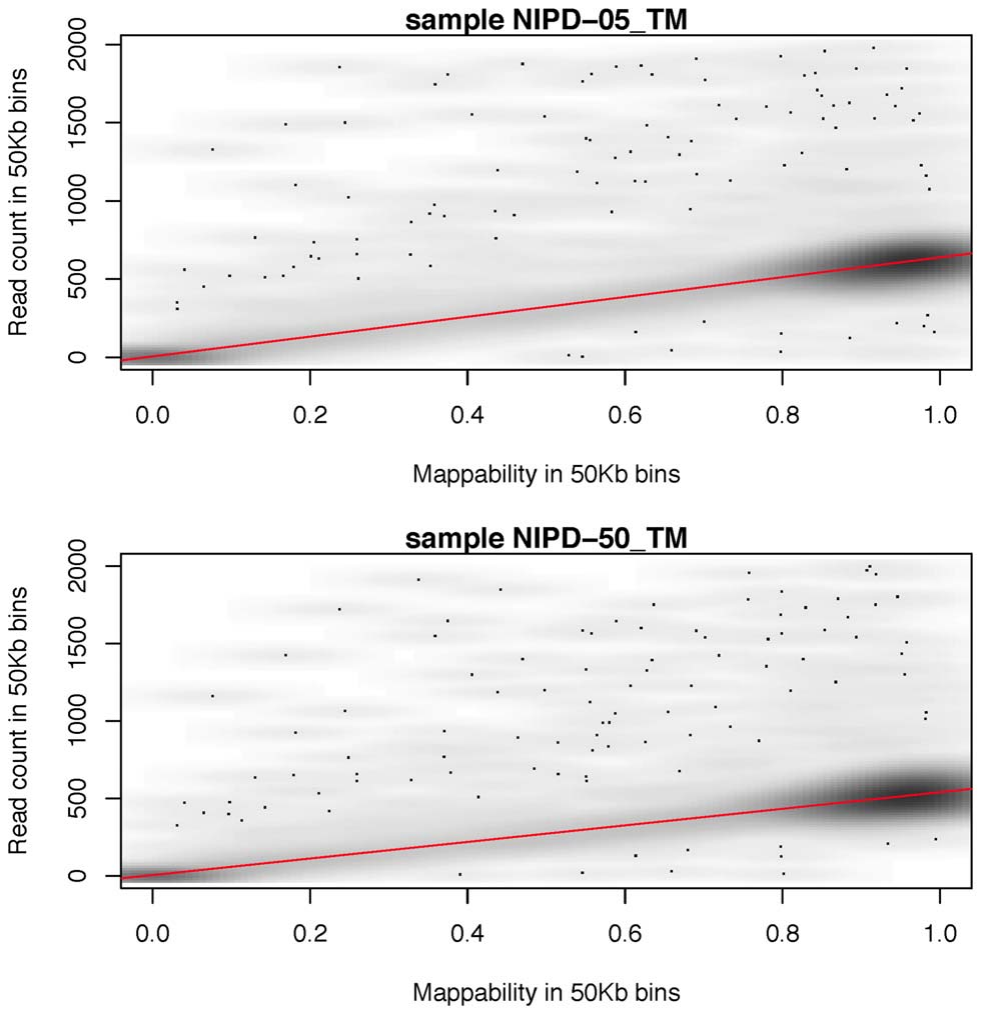
Mappability bias. The smoothed scatter plot of the mappability (as calculated by mapping the fragmented hg19 reference to itself using Novoalign) against the read count in 50 Kb genomic windows shows a linear relationship. Extreme bin counts have been removed for plotting.

The effect of mappability on the read coverage is shown in Figure 1 using two representative samples. The mapping bias in the plasma samples show no difference to the bias reported for genomic DNA in that the mappability effect was linear and therefore a reciprocal correction was suitable.

### Repeat handling

**Figure 2:**
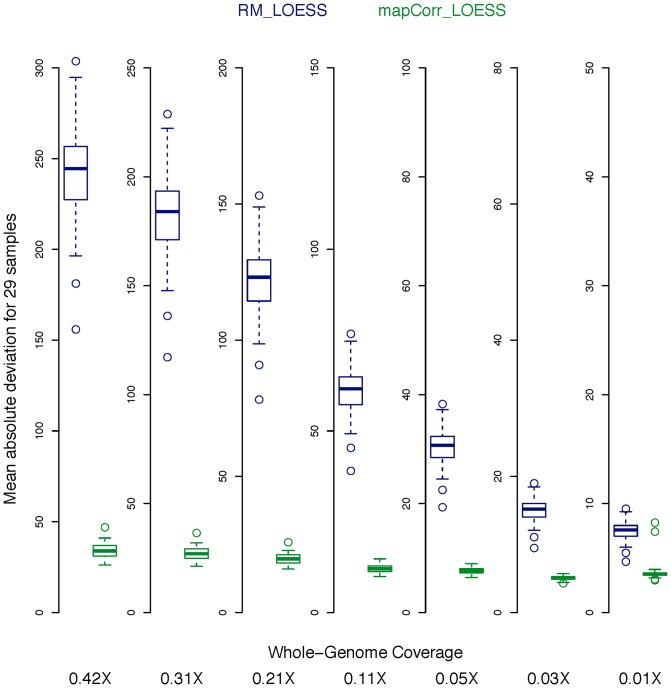
Comparison of the repeat handling methods. The prediction error (mean absolute deviation around the predicted rates) in the two repeat handling methods for 29 samples at different depths of coverage. RM_LOESS (blue) is the protocol where RepeatMasker regions are filtered and mapCorr_LOESS (green) is where mappability is quantified and read counts are corrected.

Figure 2 presents the Mean Absolute Deviation (MAD) or prediction error in data processed using the algorithms RM_LOESS and mapCorr_LOESS. MAD estimates are presented for the 29 samples at all seven coverage levels. The protocol that made use of the mappability correction (mapCorr_LOESS) consistently gave a lower error than that which made use of the method based on removing reads identified as repetitive by RepeatMasker (RM_LOESS). For coverages between 0.42X to 0.21X, mapCorr_LOESS (green) reduces error by 80% in comparison to RM_LOESS (blue) whilst for the lower sequencing depth the reduction in error is approximately 30%.

### Estimating the best GC window size for the single-position model

**Figure 3:**
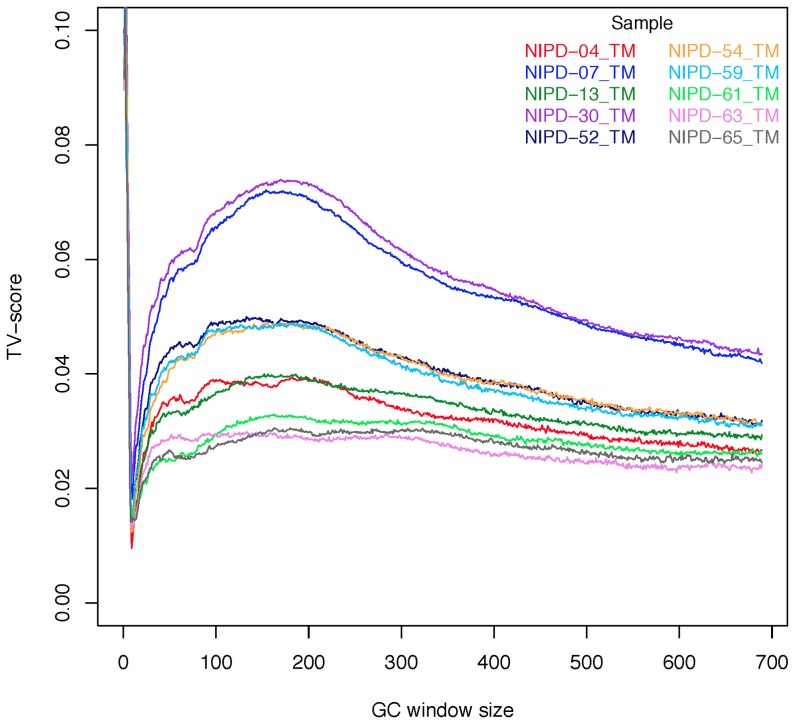
Estimating the optimal window size for GC content. The truncated plot of the scaled TV scores for 10 representative samples as the GC window size is varied. The TV score indicates the suitability of the window length used to calculate GC content. The peak at windows less than 10–250 bp stems from the wide distribution of fragment lengths in plasma. The TV score is truncated for the fragmentation peak (the range for this peak for the 29 samples is between 0.11 and 0.15).

Figure 3 shows the TV score as the GC window length is incrementally increased from one to 700 bp for 10 representative samples. All samples showed a spike for window sizes less than 10 bp, at the start of the read fragments. This is evidence for an abundance of GC dependent motifs at the fragment breakpoints. Disregarding the fragmentation effect, high TV-scores can also be seen in the range of 150–250 bp, but the function is very flat, making it difficult to determine a maximum. Similar plots for genomic/nuclear DNA derived NGS data show clear peaks at the median fragment lengths of the DNA library [37]. In contrast, plasma data indicates a range of values that is consistent with the nucleosomal lengths associated with plasma DNA fragments and the fact that DNA was not size selected in the library preparation step. When the TV scores for a sample did not indicate a clear maximum, the window size of 180 bp was chosen, which is similar to the average length of DNA wrapped around a nucleosome. The optimal GC window size selected for each sample is provided in Table S2.

### Fragmentation Effect

**Figure 4:**
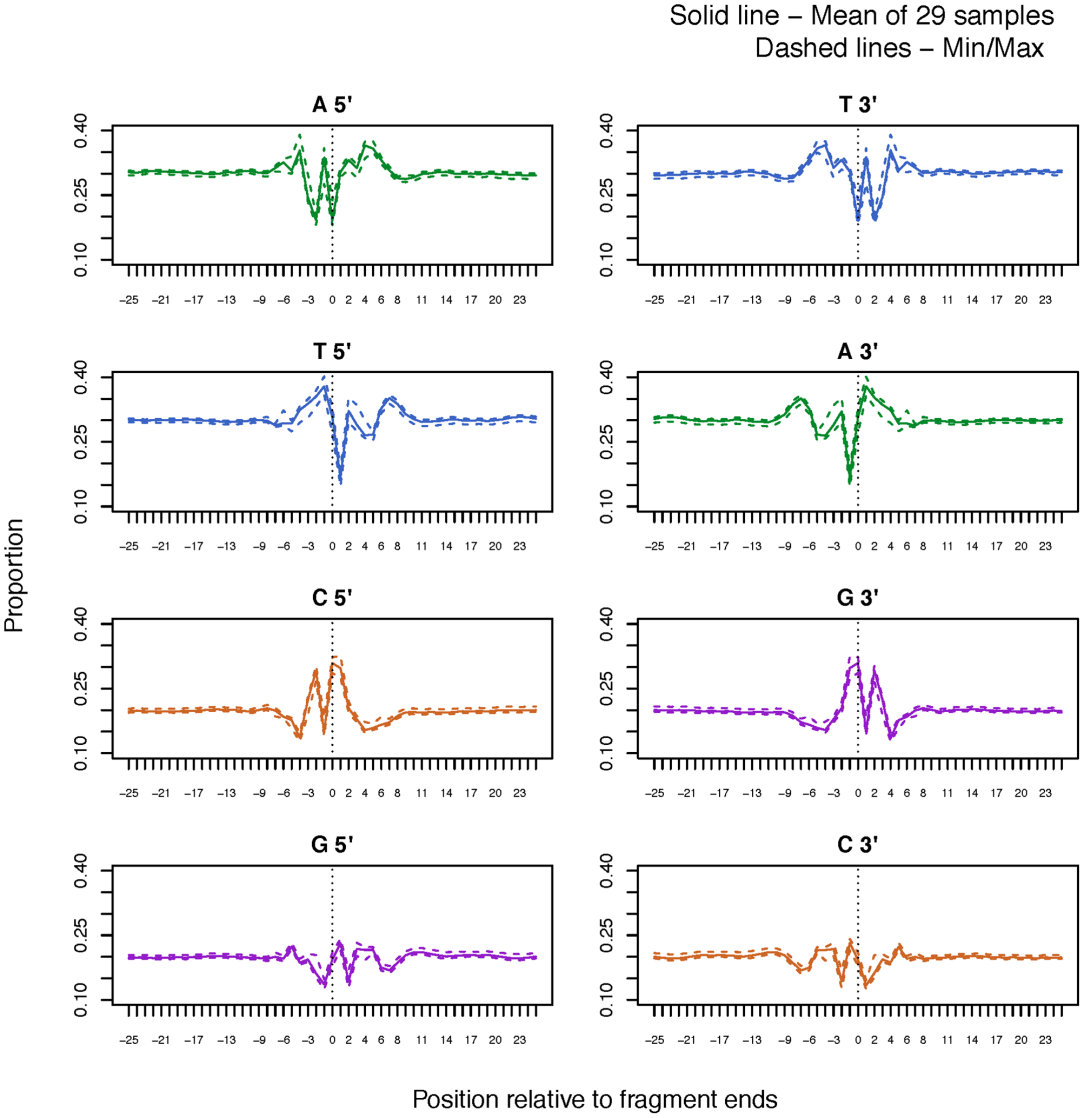
Investigating the fragmentation effect. The y-axis denotes the proportion of each nucleotide at fixed positions relative to the fragment 5′ and 3′ ends. The vertical line at 0 denotes the start of the read on each strand. The mean, minimum and maximum proportions of 29 samples have been plotted on the same graph and show that the variability between the samples is nearly negligible.

**Figure 5:**
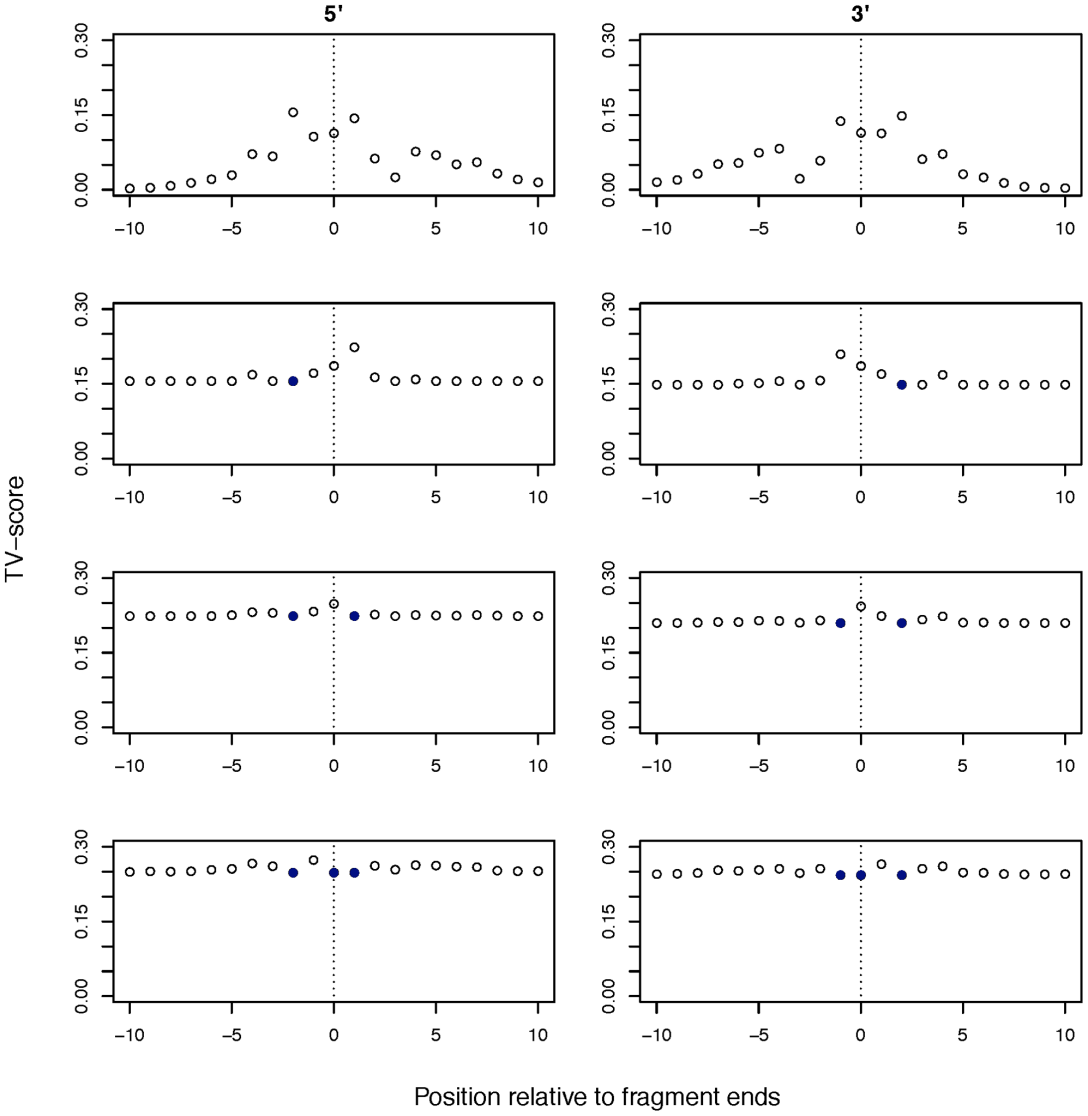
Defining a model for the fragmentation effect. The TV-score of one representative sample, conditioning on individual positions at breakpoints on the 5′ (right) and 3′ (left) strands sequentially, starting with −2 bp position (2nd row), then adding the 1st bp position (3rd row) and finally the 0th bp position (4th row). After each level of conditioning, the position that shows the highest TV score (in blue) is aggregated into the k-mer used to stratify the reads to set up the next conditioning step and the TV score is recalculated. Bias is no longer visibly location-specific after incorporating the 3-mer at positions −2,0,1. Hence, these positions can be used in a simplified model for the fragmentation bias.

The investigation of base composition around the read starts presented a strong fragmentation effect. Figure 4 shows the relative abundance of nucleotides at positions up to 25 bp on either side of the fragment breakpoint (represented as the average across all 29 samples). The nucleotides show a position-specific pattern between the bases beginning 8–9 positions before the fragment and ending 8–10 positions inside the read fragment. Guanine (G) and Cytosine (C) bases are preferentially observed at the breakpoint as indicated previously by the TV score plot. There is no evidence for a strand specific fragmentation signature since the 3′ ends of the fragments show the reverse complement of the 5′ pattern. Although the pattern extends over ∼18 positions, the position specific TV-score plots (Figure 5) show that the most influential bases can be found at positions −2, 0 and 1, with 0 indicating the start of reads. Hence, these positions can be used to simplify the motif structure for analysis purposes.

This fragmentation pattern was also represented in plasma DNA prepared using the beta ChIP-Seq library protocol optimized for low input DNA with no actual chromatin immunoprecipitation (data not shown). Furthermore, this fragmentation signature is highly replicable across technical replicates and independent samples with very little variability indicating that it is likely to be reflecting the existence of biological signal in the observed circulating cfDNA fragments.

**Figure 6:**
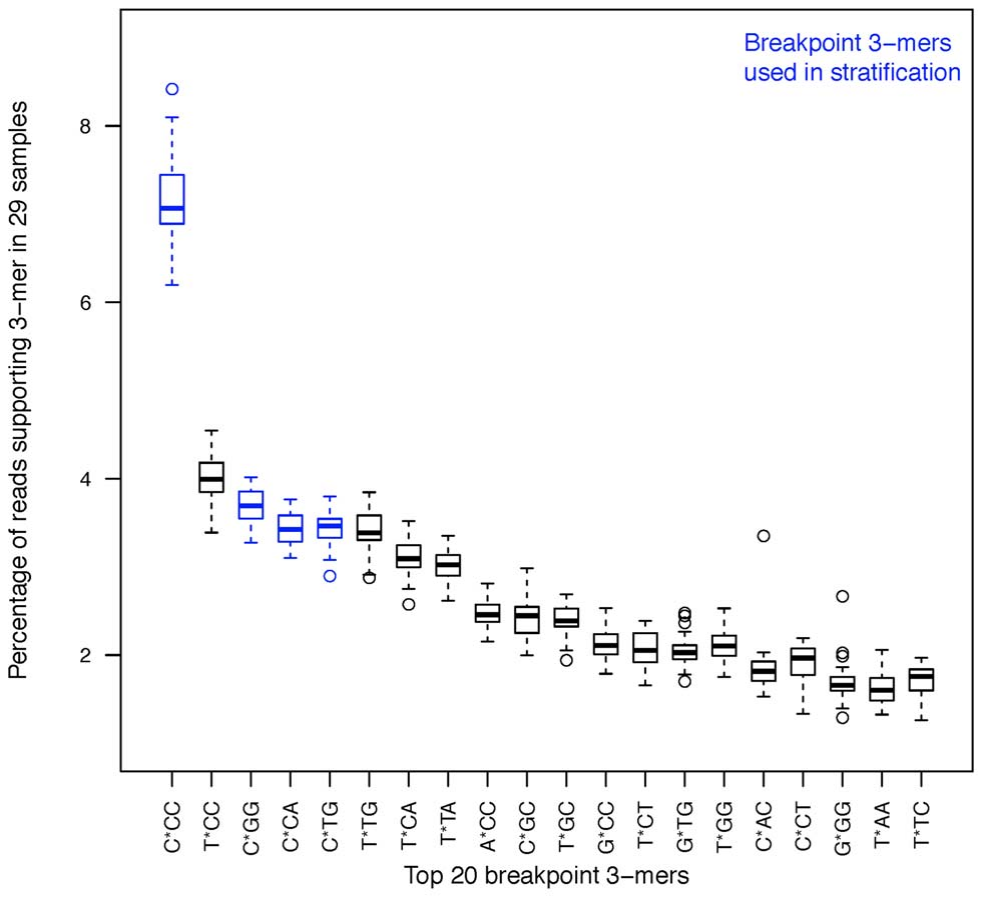
Investigating DNA motifs at the fragment breakpoints. The percentage of reads for the top twenty 3-mers at the 5′ breakpoint across all samples. Four high frequency motifs (shown in color) were grouped into one class and used in the stratification of reads. This grouping presented the divergent GC profiles shown in Figure 7.

A preference for specific motifs was apparent when reads were classed into the 64 possible 3-mers using the positions at −2,0,1 relative to the breakpoints. Figure 6 shows the proportions across the 29 samples using reads mapping to the 5′ strand. The C*CC motif was over-represented above all others with a median percentage of 7%. The motifs of T*CC, C*GG, C*CA, C*TG and T*TG were also given a higher preference (3–4.5%). The plot shows that the proportions have minimal variation between samples and the chi-square test for homogeneity using the motif proportions between the two DNA strands showed no evidence of originating from different distributions (p-values for the 29 samples ranged between 0.095 and 0.971).

**Figure 7:**
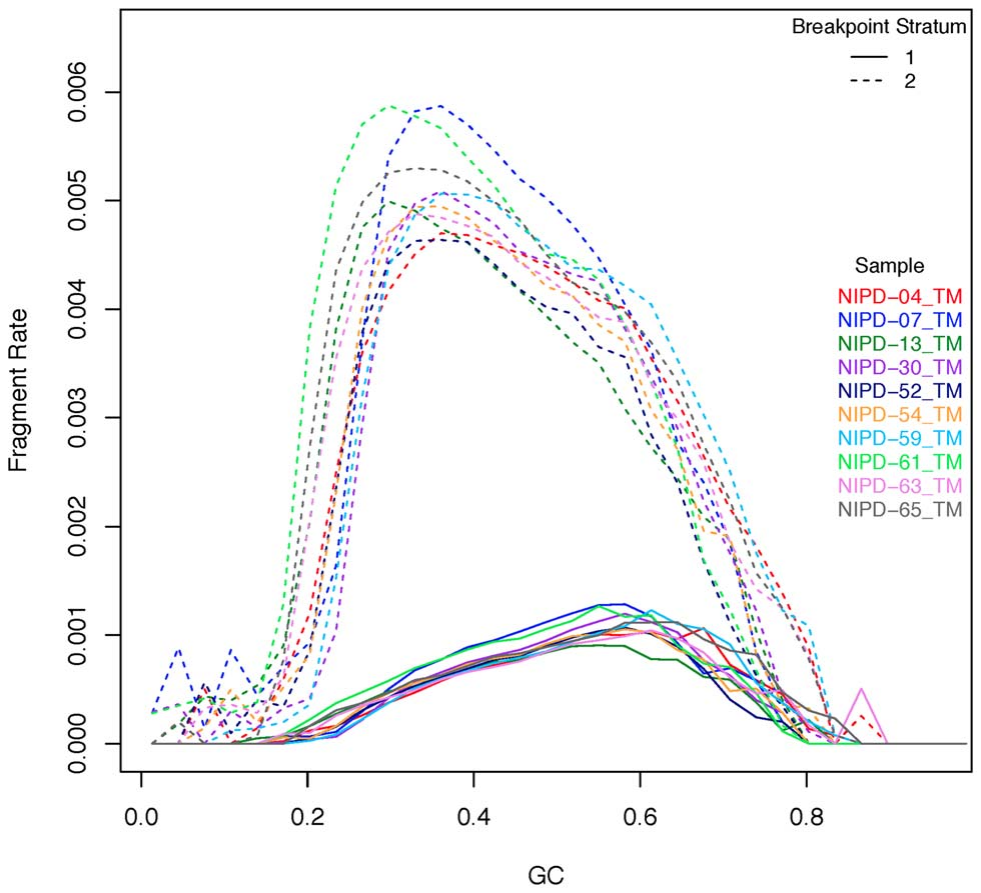
The GC bias profiles of the fragmentation motif classes. The GC profile of reads belonging to stratum 1 (reads with motifs C*CC, C*GG, C*CA and C*TG inferred from the positions −2, 0, 1 of the mapped reads) and stratum 2 (not any of these 4 motifs). Within each sample, the GC bias show different peak locations for the two sets of DNA fragments, indicating the need for separate corrections.

Examination of GC bias profiles stratified for membership of each of the eight highest frequency motifs in Figure 6 showed that only four of these (C*CC, C*GG, C*CA and C*TG) influenced the differences in TVscore. Reads were stratified according to whether they had any one of these motifs (stratum 1), or not (stratum 2), with stratum 1 constituting around 16% of data in each sample. Figure 7 shows the GC bias (mean fragment rates vs. GC content) when the single-position model was run on the stratified reads. The GC-bias curve in stratum 1 shows a shift towards higher GC content compared to stratum 2.

Looking across all 29 samples, stratum 1 has the maximum number of reads observed for 60% GC on average (standard deviation  = 0.04) while stratum 2 has the maximum number of reads observed at 37% GC (standard deviation  = 0.08). The obvious shift in the GC peaks between the fragments of the two strata was the motivation for correcting the reads of the 2 strata separately.

### Evaluation of the bias correction algorithms

**Figure 8:**
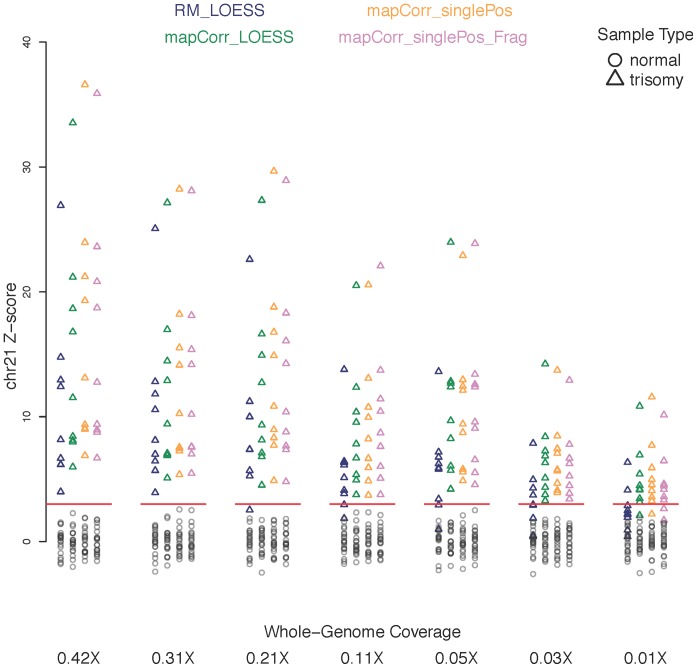
Performance of the bias correction algorithms. Chromosome 21 Z-scores for the four different methods as coverage is reduced. (Blue: RM_LOESS, Green: mapCorr_LOESS, Orange: mapCorr_singlePos, Violet: mapCorr_singlePos_ Frag). The red line denotes the diagnostic threshold of +3 for trisomy 21 detection.

Figure 8 shows the chromosome 21 Z-scores for each coverage level and bias correction protocol. Table 3 provides the discriminatory distance between the trisomy and normal Z-scores as a summary of Figure 8. Negative values of this statistic occur when the distributions of trisomy and normal Z-scores overlap eachother.

**Table 3:** Discriminatory distances for the different corrections at the 7 coverage levels.

Coverage	RM_LOESS	mapCorr_LOESS	mapCorr_singlePos	mapCorr_singlePos_Frag
0.42X	2.55	4.60	7.43	4.97
0.31X	2.67	3.20	5.04	3.88
0.21X	0.94	3.46	4.54	3.29
0.11X	0.27	2.05	2.95	2.23
0.053X	−0.19	2.69	3.40	3.36
0.026X	−1.07	1.78	2.54	2.13
0.014X	−1.07	0.52	0.94	0.28

The RM_LOESS bias correction method loses sensitivity more rapidly than all the other methods as the coverage is reduced, beginning to gain false negatives at 0.2X coverage. mapCorr_LOESS performs better than RM_LOESS at all depths. The single position GC correction performs better than a LOESS correction as it increases the separation between the trisomy and normal samples. It should be noted that the discriminatory distance of the single position protocol at 0.056X is larger than that of the RM_LOESS method at the original coverage of 0.42X. The improvement of this new protocol over the RM_LOESS method is replicated in trend in the data aligned using BWA as an alternative aligner to Novoalign (Figure S1). Stratifying by breakpoint motif does not show an improvement over the original single position model, despite showing a difference in GC profiles for reads of the two strata. These observations are further corroborated in the accuracy estimates provided in Table 4.

**Table 4:** Accuracy estimates for the different corrections at the 7 coverage levels.

Coverage	RM_LOESS	mapCorr_LOESS	mapCorr_singlePos	mapCorr_singlePos_Frag
0.42X	100%	100%	100%	100%
0.31X	100%	100%	100%	100%
0.21X	96.6%	100%	100%	100%
0.11X	93.1%	100%	100%	100%
0.053X	93.1%	100%	100%	100%
0.026X	82.8%	100%	100%	100%
0.014X	75.9%	96.6%	96.6%	93.1%

Figure S3 shows that the order of the trisomy samples (based on the Z-score) stays relatively constant as the coverage is reduced.

Despite the clear improvement seen in the mapCorr_singlePos model, we also see that not all intra-chromosomal variation is removed and that it is necessary to use reference samples via the Z-score to remove remaining bias, which cannot currently be modeled (Figure S2).

## Discussion

In the past few years there have been major advances in the area of sequencing-based non-invasive prenatal testing (NIPT). To date, a number of large-scale clinical validation studies have shown that whole-genome sequencing of circulating cell-free DNA (ccfDNA) in maternal plasma can be used to detect fetal aneuploidy with high accuracy. Published tests including those in commercial use, utilize variations of the molecular counting approach where following the mapping of sequence reads to the human reference genome, the relative numbers of fragments per chromosomes are counted to detect deviations due to the extra genetic material in the fetus.

We have demonstrated that using a more refined method for GC correction and improvements in the handling of repetitive genomic regions can lead to substantial improvements in the sensitivity of the standard statistical test for trisomy 21 detection. We would suggest that with the rapid progression of the field and a focus on the roll out of large-scale studies some of the assumptions and practices made early on in the work should no longer be perpetuated but be replaced with improved analysis techniques such as those that we have demonstrated.

The first proof-of-principle NIPT studies dealt with the ‘hard-to-map’ repeat regions of the genome by aligning the sequencing reads to the repeat masked human reference. Since 50% of the genome [35] is designated as some type of repeat, it was determined that the speed, quality and quantity of mapping was lowered when aligning directly to the masked reference and that it also lead to increased false-positives in trisomy detection [42]. Later NIPT studies opted to remove any reads in the annotated regions post-alignment (using the RepeatMasker database). Even as the sequencing-based NIPT was clinically translated and the sequencing depth was dramatically reduced for cost-efficiency the practice of using RepeatMasker persisted.

This study demonstrates the application of an alternative method for repeat handling, where the mappability of regions is calculated and read counts are corrected by these values instead of using the RepeatMasker method. Our work shows that by using this ‘soft’ approach rather than hard filtering, one can avoid unnecessarily reducing the read coverage and increasing the variation of bin counts due to uneven removal of reads. In line with our findings, a large-scale NIPT study recently reported that filtering RepeatMasker regions in moderation rather than the current severe practice, also increases the performance of NIPT [43].

To correct the GC bias we opted to use the protocol introduced by Benjamini and Speed in 2012. In their work, using genomic DNA, it was shown that a correction based on the single position model eliminates more GC bias than a LOESS correction. Since LOESS is the method of choice in whole genome NIPT, we employed the single position GC model to investigate its performance in the setting of trisomy detection using plasma DNA. This is the first instance of the application of this model in the NIPT setting.

The result of our work shows that the single-position GC correction coupled with the aforementioned mappability correction shows a significant improvement over the LOESS method with RepeatMasker by maintaining 100% sensitivity of trisomy detection over a range of decreasing depths of coverage. In particular, the latest NGS based NIPT studies, use approximately 0.2X coverage while we demonstrate 100% sensitivity down to 0.03X or 1/7^th^ the data. The discriminatory distance between trisomy and normal samples is also much higher with the new bias correction. We also show that these improvements are not aligner specific by replicating the results using a different alignment algorithm.

The performance of the methods we have demonstrated would improve further if a larger pool of reference samples were available or by using reference samples that show better matching to reduce technical variation between sequencing runs [24], [32], [44]. Another approach made possible with larger groups of reference samples, adopted by some NIPT analysis protocols, involves matching chromosomes of interest to reference chromosomes that have similar coverage variation across sequencing runs. This is one method whereby the expected read counts are adjusted to correct remaining bias after standard intra-sample adjustments for GC and repeats [31], [36]. This method is based on ad-hoc criteria and it would be desirable to identify and remove further sources of bias using justifiable approaches. Our study is carried out with a limited number of samples; hence the above methods are not applicable. By improving intra-sample bias correction strategies, we can reduce the reliance on reference samples, but as yet it is not possible to forego these entirely as we have also demonstrated.

In addition to the improvement in bias reduction, the single position model estimates corrections at the single base pair level, rather than at a binned level so it has clear advantages in terms of resolution when detecting sub-chromosomal copy number changes in the fetus. However, in an effort to benchmark our improvements against existing protocols we have not taken full advantage of these improvements. For example we bin our single base pair predictions into 50 Kb bins, using the standard protocol currently favored by researchers. We have evidence (data not shown) that further gains are possible if the size of the window for binning was reduced, analogously to Benjamini and Speed [37]. If paired-end information is available the model can also be extended to incorporate different fragment lengths as the bias stems from the GC content of the full DNA fragment. This would lead to an even more sensitive correction as plasma DNA shows a multi-modal size distribution with fetal and maternal fragments differing in lengths. We were not able to investigate the further possible gains this would make in trisomy 21 detection, as we did not have paired-end data.

We also investigated the effects due to the natural cleavage of plasma DNA using moderate coverage sequencing data, which has not been explored in depth using next generation sequencing. When using genomic DNA the input genetic material is fragmented artificially via sonication or nebulization protocols, which creates a wide array of fragment lengths. These are then size-selected depending on research specifications. In contrast, plasma DNA is fragmented by natural nuclease action and the fragments are not size selected in the library preparation for NGS.

We have shown that there are several high frequency motifs in the nucleotide frequencies spanning 8–10 positions on either side of the DNA cleavage site and that the positions on which fragment rates depend the most are the second base before the breakpoint as well as the first two bases into the fragment. We note that the cytosine rich motif that we observe at these positions has also been reported by others using cloned ccfDNA [45]. However, in an in-vitro study of the apoptotic digestion of lymphocyte chromatin, an AT-rich motif was uncovered [46], indicating that the enriched motifs we observe in our data are likely to result from a combination of biological processes.

When sequence fragments were stratified by over-represented motifs, a shift in the GC bias curve between strata was revealed, which was an indication that these fragments should be corrected separately, in two groups. However, there was no improvement over the results of the single position GC model with the mappability correction using the un-stratified data, and the stratified method performed marginally worse. This could be due to the presence of only a small subset of the reads (16%) that fall into the stratum with the enriched motifs and estimating the bias with a small number of reads at a low sequencing depth generates too much variability at the gain of little bias improvement.

The fragmentation effect seen in plasma DNA is different to that seen in genomic NGS data [37], [47]. Since these observations are highly replicable across multiple samples and across different library preparation protocols we have reason to believe that these enriched motifs reflect the existence of real biological signals. Since the enzymatic action related to plasma DNA cleavage is not fully understood, the DNA fragments may stem from multiple sources, including different nucleases with differing cleavage sites and trimming of exposed fragment ends. These various processes could dilute the fragmentation motifs and result in a highly complex mixture that we only indirectly incorporate into the proposed model. We cannot rule out the possibility that there may be some motif signal arising from technical aspects of the library preparation for ccfDNA (e.g. end-repair), but this can only be teased apart by carefully designed, deep sequencing experiments.

Whilst we have employed a better correction for GC bias in plasma DNA sequencing samples, there exist further sources of bias, which are as yet unknown, and cannot currently be corrected for within samples. The removal of these sources of bias necessitates the use of reference samples. These sources of variation need to be investigated further through deeper sequencing and analysis of plasma derived DNA. Such an analysis will also extend our biological understanding of the plasma DNA cleavage process.

## Supporting Information

Figure S1 **Chromosome 21 Z-scores for the two main bias correction protocols as coverage is reduced in read data aligned by BWA.** Blue: RM_LOESS, Orange: mapCorr_singlePos. The red line denotes the diagnostic threshold of +3 for trisomy 21 detection.
(TIFF)

Figure S2
**Reference free, intra-sample trisomy detection with the single position GC model with mappability correction.** The plot of the minimum t-statistic for each chromosome in all 29 samples, calculated from pair-wise Welsh t-tests. The dashed line corresponds to the statistic associated with 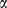
<0.001/(22×21 comparisons).(TIFF)

Figure S3 **Tracking samples across the different methods and coverage levels.** Chromosome 21 Z-scores for the four different methods as coverage is reduced. Different symbols are used to track the trisomy samples across the different coverage levels in each bias correction protocol. (Blue: RM_LOESS, Green: mapCorr_LOESS, Orange: mapCorr_singlePos, Violet: mapCorr_singlePos_ Frag). The red line denotes the diagnostic threshold of +3 for trisomy 21 detection.
(TIFF)

Table S1 Genome-wide coverage at each sampling proportion for the 29 datasets after BWA mapping and subsequent read filtering.
(DOC)

Table S2 GC window sizes for the 29 samples estimated using the TV score.
(DOC)

Text S1 Reference-free intra-sample trisomy detection.
(DOC)
