# Supplementary figures and images for: Investigating and Correcting Plasma DNA Sequencing Coverage Bias to Enhance Aneuploidy Discovery
Source: PLoS One. 2014 Jan 29;9(1):e86993. doi: 10.1371/journal.pone.0086993 (PMC3906086; doi:10.1371/journal.pone.0086993)

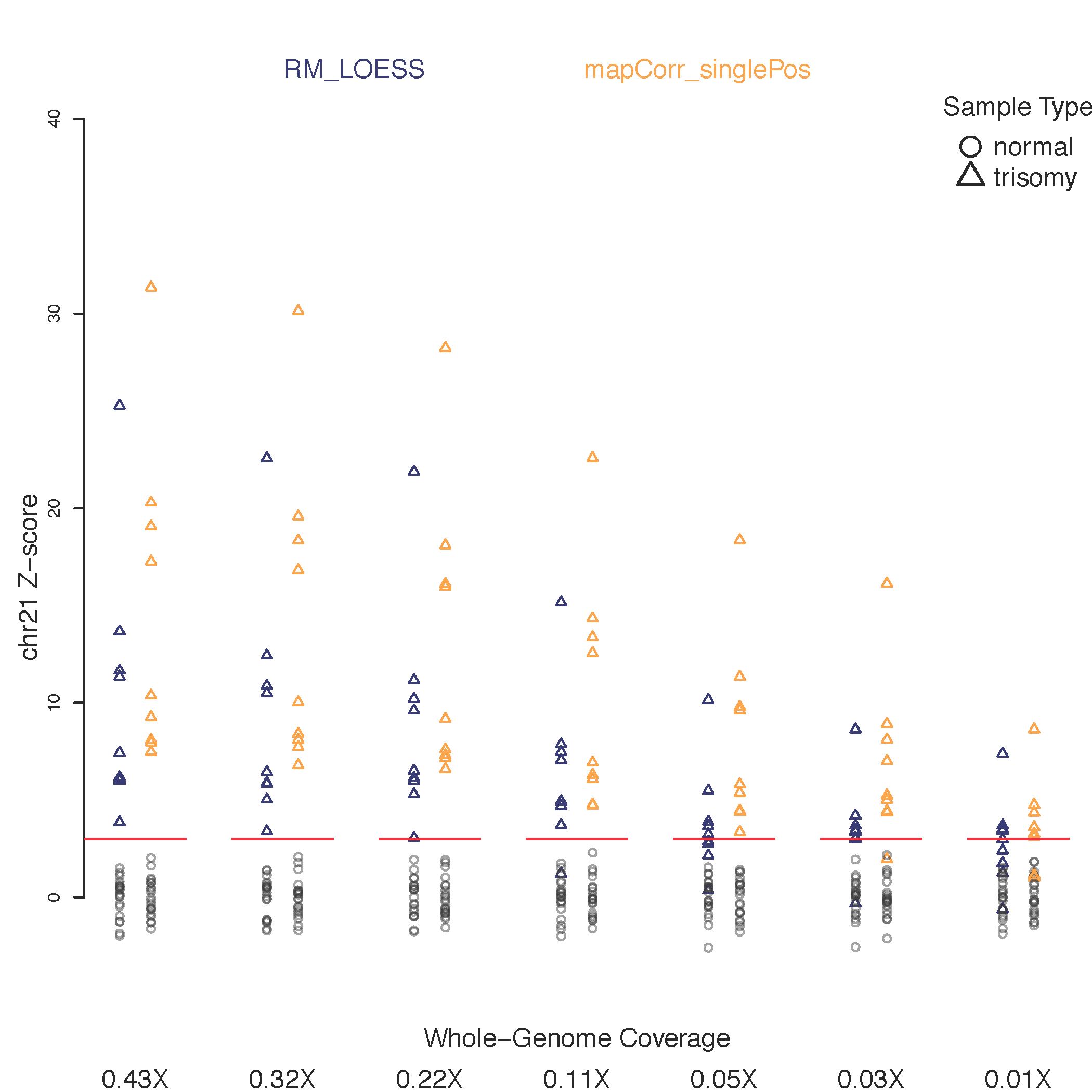

Supplement: Figure S1 — Chromosome 21 Z-scores for the two main bias correction protocols as coverage is reduced in read data aligned by BWA. Blue: RM_LOESS, Orange: mapCorr_singlePos. The red line denotes the diagnostic threshold of +3 for trisomy 21 detection. (TIFF) [file pone.0086993.s001.tiff]

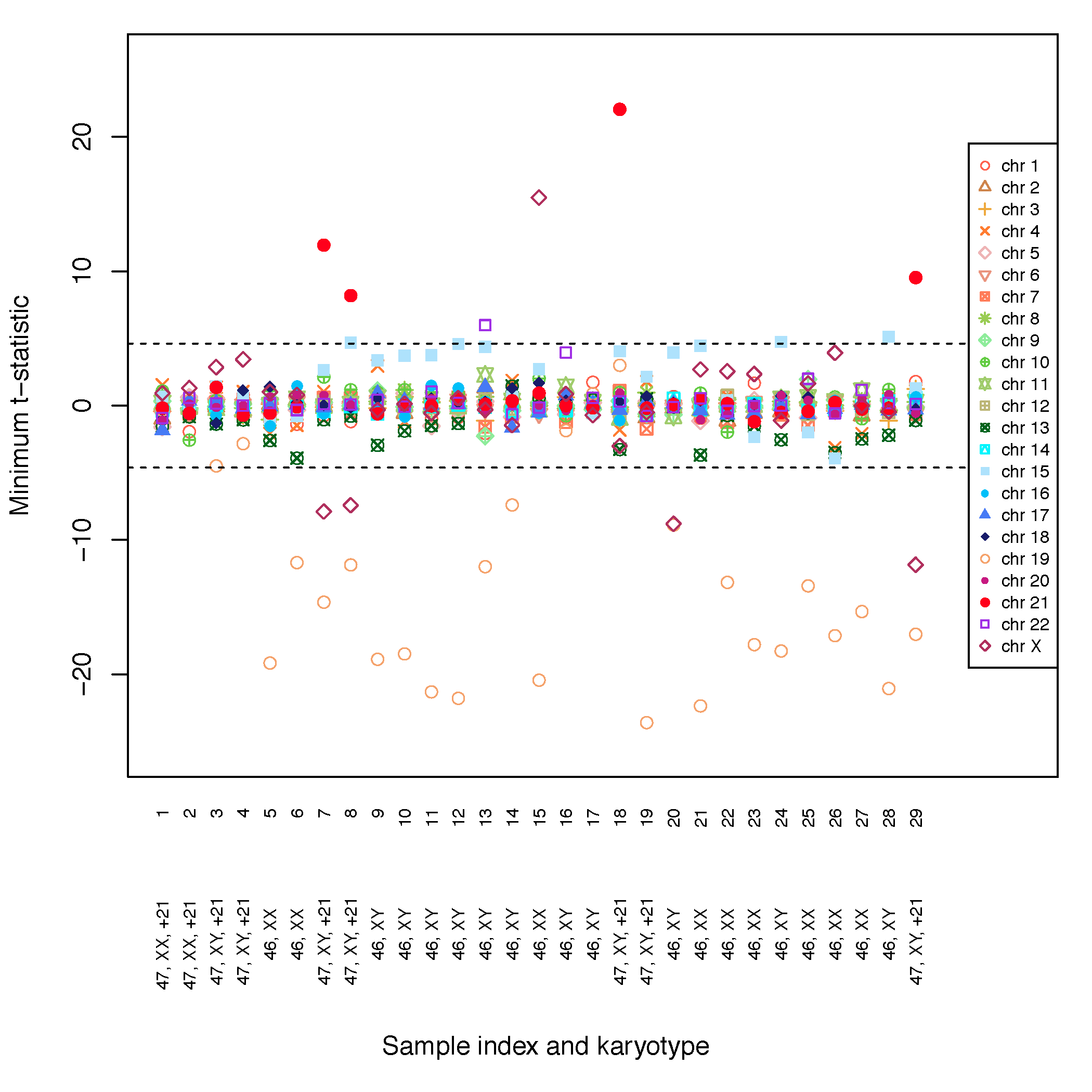

Supplement: Figure S2 — Reference free, intra-sample trisomy detection with the single position GC model with mappability correction. The plot of the minimum t-statistic for each chromosome in all 29 samples, calculated from pair-wise Welsh t-tests. The dashed line corresponds to the statistic associated with <0.001/(22×21 comparisons). (TIFF) [file pone.0086993.s002.tiff]

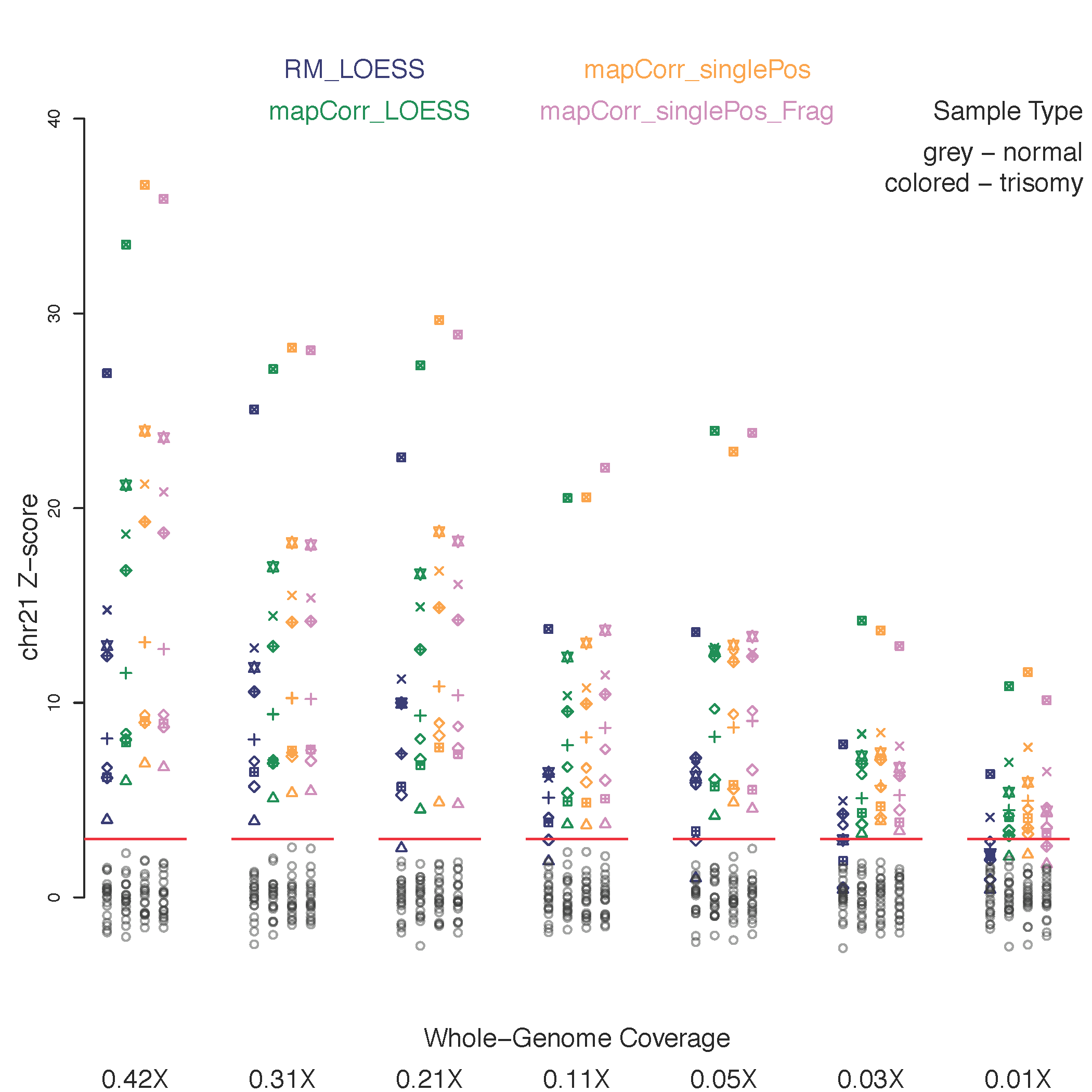

Supplement: Figure S3 — Tracking samples across the different methods and coverage levels. Chromosome 21 Z-scores for the four different methods as coverage is reduced. Different symbols are used to track the trisomy samples across the different coverage levels in each bias correction protocol. (Blue: RM_LOESS, Green: mapCorr_LOESS, Orange: mapCorr_singlePos, Violet: mapCorr_singlePos_ Frag). The red line denotes the diagnostic threshold of +3 for trisomy 21 detection. (TIFF) [file pone.0086993.s003.tiff]
