# Supplementary material for: Investigating and Correcting Plasma DNA Sequencing Coverage Bias to Enhance Aneuploidy Discovery
Source: PLoS One. 2014 Jan 29;9(1):e86993. doi: 10.1371/journal.pone.0086993 (PMC3906086; doi:10.1371/journal.pone.0086993)
Supplement: Table S1 — Genome-wide coverage at each sampling proportion for the 29 datasets after BWA mapping and subsequent read filtering. (DOC) [file pone.0086993.s004.doc]

| **Proportion of reads sampled** | **Average number of reads** | **Range** | **Mean fold coverage** |
| --- | --- | --- | --- |
| original | 26,760,848 | 19,320,529 – 33,425,147 | 0.43 |
| 0.75 | 20,166,244 | 14,546,348 – 25,206,099 | 0.32 |
| 0.5 | 13,508,445 | 9,736,346 – 16,897,014 | 0.22 |
| 0.25 | 6,790,473 | 4,892,140 – 8,500,926 | 0.11 |
| 0.125 | 3,407,498 | 2,455,664 – 4,267,322 | 0.05 |
| 0.0625 | 1,708,672 | 1,231,570 – 2,140,544 | 0.03 |
| 0.0312 | 875,682 | 631,654 – 1,097,099 | 0.01 |
