# Supplementary material for: Investigating and Correcting Plasma DNA Sequencing Coverage Bias to Enhance Aneuploidy Discovery
Source: PLoS One. 2014 Jan 29;9(1):e86993. doi: 10.1371/journal.pone.0086993 (PMC3906086; doi:10.1371/journal.pone.0086993)
Supplement: Table S2 — GC window sizes for the 29 samples estimated using the TV score. (DOC) [file pone.0086993.s005.doc]

| **Sample Index** | **GC Window Size** | **Sample Index** | **GC Window Size** | **Sample Index** | **GC Window Size** |
| --- | --- | --- | --- | --- | --- |
| 1 | 208 | 11 | 153 | 21 | 206 |
| 2 | 211 | 12 | 159 | 22 | 169 |
| 3 | 198 | 13 | 192 | 23 | 164 |
| 4 | 186 | 14 | 198 | 24 | 180 |
| 5 | 191 | 15 | 156 | 25 | 219 |
| 6 | 184 | 16 | 185 | 26 | 171 |
| 7 | 160 | 17 | 205 | 27 | 180 |
| 8 | 175 | 18 | 180 | 28 | 164 |
| 9 | 180 | 19 | 177 | 29 | 168 |
| 10 | 180 | 20 | 134 |  |  |
