# Supplementary material for: Investigating and Correcting Plasma DNA Sequencing Coverage Bias to Enhance Aneuploidy Discovery
Source: PLoS One. 2014 Jan 29;9(1):e86993. doi: 10.1371/journal.pone.0086993 (PMC3906086; doi:10.1371/journal.pone.0086993)
Supplement: Text S1 — Reference-free intra-sample trisomy detection. (DOC) [file pone.0086993.s006.doc]

**Reference-free intra-sample trisomy detection**

The capability of the mapCorr_singlePos bias correction protocol in detecting trisomy in the absence of controls was investigated. This analysis was undertaken to assess any residual inter-chromosomal bias after the aforementioned coverage corrections.

This protocol was carried out on the Novoalign mapped reads of all 29 samples at their original coverage as described in Materials and Methods. For this analysis, the predictions were binned for genomic windows of 300bp length for better correction of local biases and a mappability threshold of 0.9 was employed.

To determine whether the copy number of a chromosome within a patient sample deviated from the normal, every chromosome was compared against all others (except for chrY) in pairwise Welch’s t-tests for difference in means using the binned counts after correction. The minimum t-statistic, or first order t-statistic was used to assess significance. It was required that chromosomes be different from all others at significance level α < 0.001 (after a Bonferrroni correction was applied to account for multiple testing) to be considered over or under-represented. It was expected for trisomy samples to show high t-statistics for chromosome 21 and for male samples to show low chromosome X values.

Supplementary Figure S2 depicts the results for this analysis. It can be seen that under this stringent criteria, only 4 out of 9 trisomy samples were detected and only 4 of 18 male samples were correctly identified. The behavior of chromosomes such as 19 and 15 show that not all biases between the chromosomes have been corrected. Chromosome 19 for example, has the highest GC and genomic repeat content in the genome leading to the lowest read count (adjusted for length) among the chromosomes.

This analysis was based on the proof of principle study by Fan and Quake in 2010. Similar to our observations, they noted the persistence of coverage bias and carried out a further inter-sample correction of the bin counts by using the read distributions of control datasets. This shows that the bias corrections are not yet sensitive enough to carry out reference-free trisomy detection and that the sources for the uncorrected bias need further investigation.

**References**

Fan H, Quake S (2010) Sensitivity of noninvasive prenatal detection of fetal aneuploidy from maternal plasma using shotgun sequencing is limited only by counting statistics. PLoS ONE 5.
